# Prevalence and risk factor of diabetes among the elderly people in West Bengal: evidence-based LASI 1st wave

**DOI:** 10.1186/s12902-023-01421-3

**Published:** 2023-08-11

**Authors:** Ujjwal Das, Nishamani Kar

**Affiliations:** 1 Dept. of Geography in, Rajiv Gandhi University, Doimukh, Arunachal Pradesh India; 2 Dept. of Geography, Fakir Mohan University, Balasore, Odisha India

**Keywords:** Diabetes, Dietary pattern, Logistic regression, Marital status, Rural-urban ratio, Urbanization

## Abstract

**Background:**

The complication of Diabetes is one of the important health issues among the older adult population in any region. The higher risks of diabetes prevalence among older adult people in the countries was due to social-cultural changes such as increasing urbanization, dietary changes, without physical activity, and unhealthy lifestyle behavior. The present study examines the prevalence and associated risk factors of diabetes among older adults in the state of West Bengal.

**Methods:**

The first wave of the Longitudinal Ageing Study in India 2017-18 was used to achieve the study objectives. Descriptive statistics with multinomial logistic regression models were used to carry out crude and adjusted odds ratios with 95% confidence intervals and examine the associated risk factors of diabetes prevalence among older adults.

**Results:**

The findings of the study indicate that the overall prevalence of diabetes among the study participants was found to be 12.4% which was significantly higher in urban areas (19%) compare to rural areas (6%). The socio-economic and bio-demographic factors like educational status, richest background family, marital status, obesity, and family history of diabetes were significantly associated with higher risks of diabetes prevalence among the older adult population in West Bengal. The risks of diabetes in the richest adult people were significantly higher than in the poorest adult people (OR = 2.78; 95% CI: 1.974–3.917). The higher risks of diabetes mellitus among the richest wealthy people are because of lifestyle behavior, smoking, and tobacco consumption respectively.

**Conclusion:**

The study needs to policy and awareness program to reduce economic inequality and prevention of diabetes care and treatment-seeking behavior, especially for the older adult population in West Bengal.

## Introduction

Diabetes is one of the non-communicable diseases which lead to major public health challenges and economic loss throughout the world and it is predicted that in the year 2030, it’s become the seventh leading cause of death in the world. According to the report of the International Diabetes Federation, in 2015, around 415 million people are suffering from diabetes mellitus and it is expected that it will rise to 642 million by 2040 [1]. Diabetes Mellitus in low-middle-income countries is one of the tremendous public health issues and challenges and it accounts for 75% of the population incidence of diabetes every year. The incidence of diabetes has more coverage among urban residents but the recent literature also suggested that diabetes incidence is also increasing among rural residents [2]. In developing countries like Asia, is the main source for arising of epidemics of diabetes and it contributes to more than 60% of the global burden of diseases in diabetes mellitus [1, 3]. The high prevalence of diabetes mellitus in Asian countries is due to rapid socio-economic changes including economic wealth status, lifestyle behavior, the unprecedented growth of urbanization, and nutritional transaction [4].

The prevalence of diabetes in India is responsible for a main global s burden of diseases. It is estimated that in India 77 million people suffer from diabetes, thus the country is considered the second highest diabetic capital in the world followed by China [5]. It is projected that the diabetes burden to be 101 million in 2030 and 134 million in 2045 [6]. In 2013, 65.1 million people were suffering from diabetes in the age group between 20 and 79 years and this number is predicted to rise to 109 million by 2035 [7, 8]. The prevalence range of diabetes mellitus in India is 5–17% per year with the higher level found in the southern part of the country [9–12]. For example, Kerala is the unique southern state where the highest prevalence of diabetes has been found followed by the eastern state of West Bengal. A growing number of studies suggested that larger regional and unequal distribution of socio-economic diversity among the people is the main reason for higher diabetes prevalence in India [13–15]. Diabetes prevalence was highest among the older adult age group particularly in the age above 60 years because of the increasing number of the aging population, extreme growth of urbanization, health, and lifestyle behavior with western dietary patterns, and without physical activities. The earlier evidence mentioned that the rising prevalence of overweight/obesity is one of the important leading causes of diabetes in the country [16]. In addition in India, a high prevalence of metabolic cardiovascular risk factors has been found among clinic-based diabetes patients. The study found that patients with T2 diabetes mellitus 53% have a higher risk of cardiovascular diseases and this prevalence was higher among males than females (55.6% vs.50.7%) [17].

Furthermore, the out-of-pocket expenditure and delivery costs in healthcare are one of the major challenges in the private sector in India [18]. An earlier report suggested that the contribution of public health care in India was lower as compared to any other region, from the report in 2012, suggested that the South East Asia region contributed an average of 52% of health expenditure, while the Indian government contributed only 33% of total health expenditure [19]. Notwithstanding, In India shared only 4% of gross domestic product (GDP) in health care expenditure which was equivalent to the average South East Asia region [20]. Thus increasing economic inequality has widened the gap in diabetes mellitus in the place of residence. Whereas in rural areas self-reported prevalence of diabetes was lower (3.1%) compared to urban areas (7.3%) [21].

The growing number of literature suggested that older adult diabetes is substantial morbidity which is the leading cause of macro- and micro-vascular complications, higher mortality, reduced functional status, and increased risk of institutionalization [22, 23]. Hence, it is necessary to understand the burden and risk factors of diabetes and the need for some policies and programs for the specific measurement of diabetes prevalence in the older adult population. Only have few studies in West Bengal have determined the prevalence of various cardiovascular risk factors in patients with diabetes. The cross-sectional study on rural West Bengal by Barik et al., (2016), found that the prevalence of diabetes and pre-diabetes among adults > 18 years was 2.95 and 3.34% respectively [11]. In another study, Little et al. reported prevalence rate of diabetes type 2 was higher among the adult population around 10.8% in the age group below 19 years in the rural part of south India [9]. This study aimed to investigate the prevalence and risk factors of diabetes among the older adult population in the state of West Bengal.

## Materials and methods

The present study data has been used from the first wave of the Longitudinal Ageing Study in India (LASI), which was conducted in 2017-18 [24]. LASI is the first’s longitudinal ageing survey data in India; it provides information on economics, health, different healthcare policies, and social drivers of population aging in India. Total of 72,000 samples of the old age group aged 45 and above were surveyed in India’s state and union territories in the first wave of LASI data in 2017-18. The survey was done through the multistage stratified cluster sampling method. Where in rural areas data were collected through a three-stage sampling method and in urban areas a four-stage method. In the initial stage, Primary Sampling Unit (PSU) was selected, after that villages were selected in rural areas and wards are selected in the urban areas (LASI, 2020). In the third stage from the rural areas, families were chosen in various communities and randomly chosen from the Census Enumeration Block (CEB) from the urban areas respectively. And finally, Households were chosen from each CEB in the residence (LASI, 2020). The present study covered a total of 3933 older aged people 45 years and above (rural 1971 and urban 1962).

### Variable description

#### Dependent variable

The present study focuses on self-reported Diabetes. Various questions such as the health problems of individuals were asked, including ‘Has any health professional ever diagnosed you with Diabetes or high blood sugar? Therefore main dependent variable utilized for the present study is ‘Diabetes’ among the aged people 45 years and above. It is coded “1 = Yes if respondents are ever had diagnosed with diabetes otherwise it’s “0 = No”.

#### Independent variable

As established in the reviewed literature, several other socio-demographic and behavioral variables have also been used for a significant effect on diabetes prevalence among older adult people in the analysis [23, 25–29]. The Operational definitions of this variable include bio-demographic variables like sex of the respondents (male; female), age (45–54, 55–64, 65–74, and 75 + years), socio-economic variables including place of residence (rural; urban), religion (Hindu; Muslim; Christian and others), caste (SC; ST; OBC and others), marital status (Currently married; Widowed and others), education (no education; primary; secondary and higher secondary), working status (currently working, not working currently and never worked), MPCE (Monthly Per Capita Expenditure) quintile (poorest; poorer; middle; richer and richest).

The lifestyle behavioral factors included in the study ever had consumed any alcoholic beverages (yes; no), ever used smokeless tobacco including chewing tobacco, gutka (yes; no), and ever had smoking such as beedi, cigarette, and others (yes; no). The response rate is yes as codded 1 and no codded as 0.

### Statistical methods

Descriptive statistics such as frequency and percentage were used for the present study. To examine the association between outcome and explanatory variables chi-square test was used in the study. The binary logistic regression analyses were used for identifying the odd ratios (ORs) for both crude and adjusted between outcome variable (i.e., diabetes) and explanatory variables (i.e., age, sex, marital status, education, religion, caste, wealth status, working status, alcohol, tobacco, and smoking consumption) with 95% confidence intervals in the study. All the analysis has been done by statistical software STATA version 14.2 respectively.

## Results

**Table 1:** Sample distribution of diabetes prevalence among older adult people in West Bengal

Background Characteristics	Sample	Percentage
	(N)	(%)
**Age (years)**		
45–54	1,837	45.93
55–64	1,042	26.49
65–74	671	17.06
75+	383	9.74
**Sex**		
Male	1,620	41.19
Female	2,313	58.81
**Residence**		
Rural	1,971	50.11
Urban	1,962	49.89
**Marital Status**		
Currently married	2,982	75.82
Widowed	811	20.62
Others	140	3.56
**Education**		
No Education	1555	39.55
Primary	561	14.27
Secondary	1001	25.46
Higher Secondary	816	20.73
**Religion**		
Hindu	3,122	79.38
Muslim	754	19.17
Christian	19	0.48
Others	38	0.97
**Caste**		
SC	971	24.77
ST	172	4.39
OBC	433	11.05
**Working Status**		
Currently working	1754	44.64
Not working currently	833	21.2
Never Worked	1,346	34.16
**MPCE Quintile**		
Poorest	691	17.57
Poorer	943	23.98
Middle	869	22.1
Richer	811	20.62
Richest	619	15.74
**Alcohol Consumption**		
Yes	461	11.79
No	3472	88.21
**Tobacco Consumption**		
Yes	3157	80.28
No	775	19.72
**Smoking Consumption**		
Yes	1467	37.51
No	2444	62.49
**Total (N)**	**3933**	

**Source**: Authors’ own calculation using a longitudinal aging survey of India (2017–18).

Table 1 shows the sample distribution among older adult people with sociodemographic and behavioral characteristics. A total of 3933 participated in the state of West Bengal, among them 1620 (41.19%) are males and 2313 females (58.81%). Their age ranged from 45 years to 65 years and above. The majority of the study population was aged 45–54 years (45.93%). Among them, 49.9% of the older adult belonged to urban residences and 50% belonged to rural residences respectively. Overall, 75.82% of the participants were currently married and 39.55% had no formal education. In this state majority of the household belonged to the poorest to middle economic background family and only 15.74% belonged to the richest quintile respectively. Looking into behavioral characteristics percentage of substance alcohol, smoking, and tobacco consumption among older adult were 11.79%, 37.51%, and 80.28% and 80.28% respectively.

**Table 2:** The percentage of diabetes prevalence among the elderly by different background characteristics, in West Bengal

**Background Characteristics**	Yes	No	χ2 (df), *P*
**Age (years)**			
65–74	18.03	81.97	
75+	13.61	86.39	
**Sex**			
Male	14.06	85.94	6.83 (1)
Female	11.26	88.74	*P* = *0.009*
**Residence**			
Rural	6.2	93.8	140.13 (1)
Urban	18.66	81.34	*P* = *0.000*
**Marital Status**			
Currently married	12.48	87.52	0.78 (2)
Widowed	12.58	87.42	*P* = *0.676*
Others	10	90	
**Education**			
No Education	7.86	92.14	99.06 (3)
Primary	10.71	89.29	*P* = *0.000*
Secondary	12.7	87.3	
Higher Secondary	21.95	78.05	
**Religion**			
Hindu	12.68	87.32	2.71 (3)
Muslim	11.55	88.45	*P* = *0.439*
Christian	15.79	84.21	
Others	5.26	94.74	
**Caste**			
SC	9.48	90.52	26.21 (3)
ST	4.65	95.35	*P* = *0.000*
OBC	11.11	88.89	
Others	14.38	85.62	
**Working Status**			
Currently working	9.43	90.57	30.07 (2)
Not working currently	16.63	83.37	*P* = *0.000*
Never Worked	13.73	86.27	
**MPCE Quintile**			
Poorest	7.84	92.16	53.99 (4)
Poorer	10.72	89.28	*P* = *0.000)*
Middle	10.02	89.98	
Richer	15.72	84.28	
Richest	19.12	80.88	
**Alcohol Consumption**			
Yes	11.71	88.29	0.233 (1)
No	12.5	87.5	*P* = *0.629*
**Tobacco Consumption**			
Yes	9.03	90.97	7.89 (1)
No	16.77	83.23	*P* = *0.005*
**Smoking Consumption**			
Yes	10.22	89.78	10.26 (1)
No	13.71	86.29	*P* = *0.001*
**Overall Diabetes**	**12.41**	**87.59**	

**Source**: Author’s own calculation using a longitudinal aging survey of India (2017–18).

**Fig. 1:**
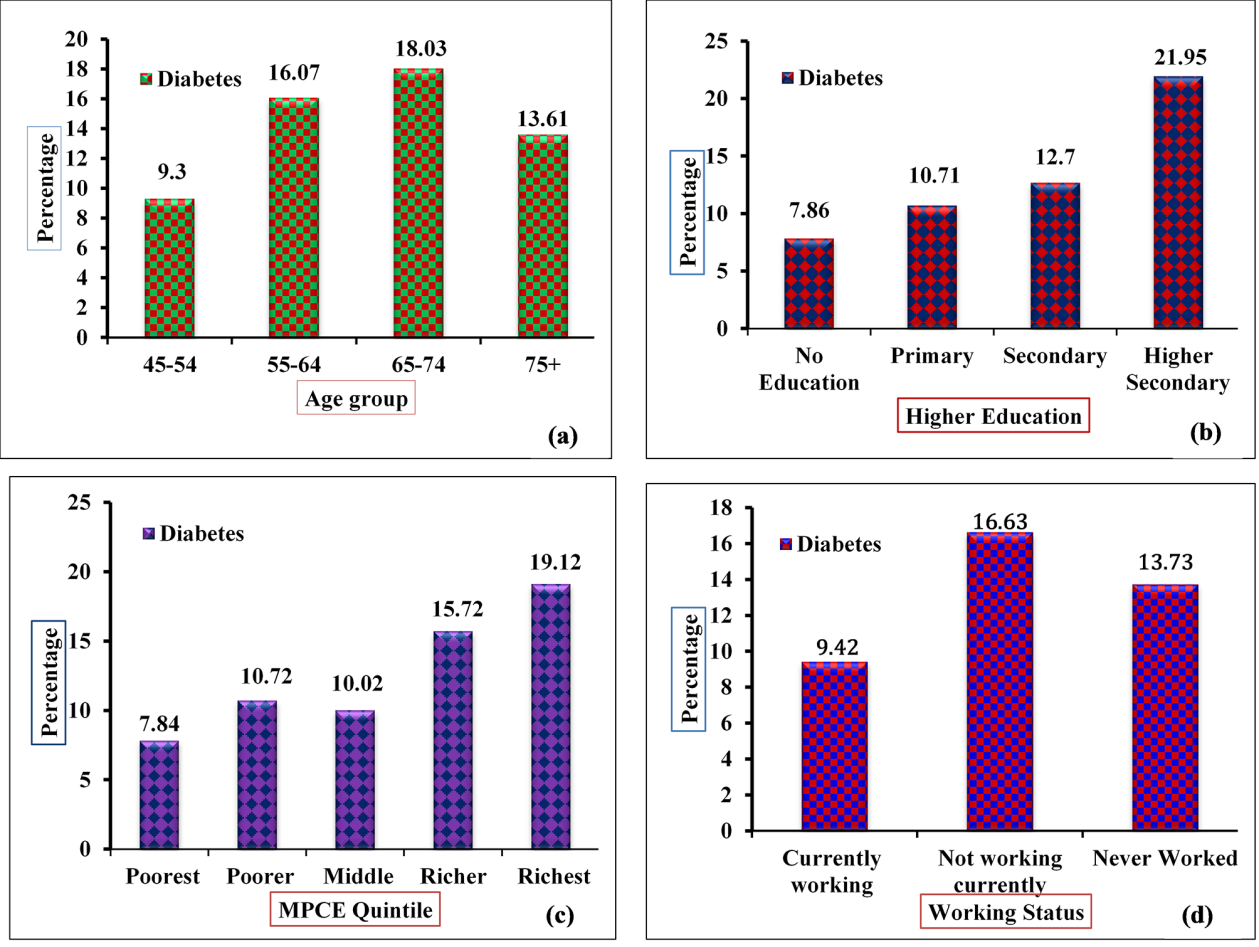
Prevalence of diabetes by different background characteristics (**a**) Age Group; (**b**) Higher Education; (**c**) MPCE Quintile; (**d**) Working Status

Table 2 depicts the prevalence of diabetes by different socioeconomic and demographic characteristics among older adult people in West Bengal. It was found the highest diabetes prevalence was in the age group of 65–74 years among the older adult. The overall prevalence of diabetes among older adult people was found to be higher in urban areas as compared to rural (18.66% vs. 6.20%) areas with significant levels. The prevalence of diabetes among males was higher as compared to females (14.06% vs. 11.26%). Married people had a significantly higher prevalence of diabetes than widowed. The highest-educated older adult groups of people had a higher prevalence of diabetes as compared to their non-education and less-educated counterparts. Older adult people who had not currently worked had a higher prevalence of diabetes as compared to those who had currently worked in any kind of activity. The study shows (Fig. 1) adult people from a higher economic background family have a higher prevalence of diabetes as compared to lower economic background family. The older adult people who belonged to the poorest background family 7.84% had diabetes and for those have belonged to the richest economic background family the prevalence of diabetes was 19.12% respectively. The prevalence of diabetes with lifestyle behavioral factors was less significant as compared to the other factors.

**Table 3:** Unadjusted and adjusted odds ratio in diabetes prevalence by selected background characteristics in West Bengal

Background characteristics	Unadjusted Odds Ratio			Adjusted Odds Ratio			
	OR	S.E	95% CI	OR	S.E	95% CI	Marginal Effects
**Age**							
45®							
45–54	2.12***	0.49	1.339–3.344	2.49***	0.61	1.538–4.039	0.054
55–64	3.96***	0.91	2.522–6.203	4.85***	1.20	2.983–7.880	0.123
65–74	4.54***	1.07	2.861–7.215	5.00***	1.32	2.974–8.394	0 0.126
75+	3.25***	0.85	1.953–5.422	3.62***	1.09	2.010–6.529	0 0.089
**Sex**							
Male®							
Female	0.78***	0.08	0.641–0.939	0.74**	0.12	0.539–1.005	-0.030
**Marital Status**							
Currently married®							
Widowed	1.01	0.12	0.798–1.275	0.84	0.12	0.627–1.124	-0.017
Others	0.78	0.22	0.444–1.368	0.59*	0.18	0.329–1.060	-0.046
**Education**							
No Education®							
Primary	1.41**	0.23	1.017–1.949	1.41**	0.25	0.997–1.993	0.031
Secondary	1.71***	0.23	1.313–2.218	1.45***	0.22	1.074–1.958	0.034
Higher Secondary	3.30***	0.42	2.572–4.229	1.85***	0.32	1.329–2.588	0.061
**Place of Residence**							
Rural®							
Urban	3.47***	0.38	2.798–4.307	3.30***	0.42	2.576–4.230	0.113
**Religion**							
Hindu®							
Muslim	0.90	0.11	0.702–1.151	1.59***	0.24	1.184–2.138	0.050
Christian	1.29	0.81	0.374–4.449	1.61	1.10	0.422–6.136	0 0.051
Others	0.38*	0.28	0.092–1.594	0.85	0.66	0.183–3.920	-0.015
**Caste**							
SC®							
ST	0.47**	0.18	0.222–0.977	0.65	0.26	0.286–1.437	-0.042
OBC	1.19	0.22	0.825 - 1.725	0.93	0.19	0.622–1.397	-0.008
Others	1.60***	0.20	1.256–2.045	0.78*	0.11	0.583–1.035	-0.026
**Working Status**							
Currently working ®							
Not working currently	1.92***	0.24	1.503–2.444	1.57***	0.23	1.179–2.098	0.042
Never Worked	1.53***	0.17	1.224–1.913	1.91***	0.30	1.400–2.609	0.064
**MPCE Quintile**							
Poorest®							
Poorer	1.41**	0.25	0.999–1.996	1.33*	0.25	0.926–1.920	0.024
Middle	1.31*	0.24	0.918–1.869	1.22	0.23	0.838–1.777	0.016
Richer	2.19***	0.38	1.567–3.070	1.81***	0.34	1.257–2.616	0.056
Richest	2.78***	0.49	1.974–3.917	2.28***	0.45	1.556–3.353	0.083
**Alcohol Consumption**							
Yes®							
No	1.08	0.17	0.80–1.46	1.04	0.19	0.722–1.486	0.003
**Tobacco Consumption**							
Yes®							
No	2.03***	0.52	1.229–3.352	1.27*	0.17	0.984–1.645	0.027
**Smoking Consumption**							
Yes®							
No	1.40***	0.15	1.142–1.711	1.28	0.36	1.262–3.383	0.023

**Source**: Authors own calculation using a longitudinal aging survey of India (2017–18).

***p < 0.01 **p < 0.05 *p < 0.1; ® Reference category.

Table 3 shows the unadjusted and adjusted odds ratio in diabetes prevalence by socio-economics and demographic factors among the older adult people in the study. The univariate level result shows the crude odds ratio for diabetes had increased with increasing the age up to 74 years. A similar pattern was also observed in the adjusted odds ratio by socio-economic and demographic variables. The sex-wise difference was found females were at lower risk of diabetes prevalence as compared to males as reference category with a statically significant 95% confidence interval (OR = 0.78; 95% CI 0.641–0.939). The older adult people with the highest education level 3.30 times for unadjusted and 1.85 times for adjusted higher risks of diabetes prevalence as compared to those who had no formal education or less education with statically significant (OR = 3.30; 95% CI 2.572–4.229), OR = 1.85; 95% CI 1.329–2.588). The risks of diabetes in urban residents were higher than the rural residents with a 95% statistically significant level (OR = 3.47; 95% CI 2.798–4.307). Considering in wealth status of the family, both adjusted and unadjusted odds for diabetes showed that the richest older adult people had a higher risk of diabetes as compared to older adult people who were belonging to the poorest family respectively (OR = 2.78;95% CI 1.974–3.917). Thus it indicates that respondents of higher prevalence of diabetes with higher economic background family because they were never worked or currently not working in any kind of activities. Lifestyle behavioral factors such as alcohol, tobacco, and smoking were not significantly associated with adjusted diabetes prevalence in the study.

## Discussion

The large-scale longitudinal aging study documented the prevalence of diabetes among the older adult population (> 45 years) in West Bengal of India, and the result suggested that the prevalence of diabetes is strongly associated with the bio-demographic, socio-economic, and lifestyle behavioral factor. The present study shows the majority of the population is aged 45–54 years (46%). Among the total older adult population, 49% belonged to rural residences and 16% of the population belonged to the richest economic background family. Coming to the main objectives of the study, the result showed that the overall prevalence of diabetes among the study participant was 12.4%. And the prevalence rate of diabetes in urban areas was higher at 19% than the rural areas at 6% respectively. However, the prevalence of diabetes was found to be much lower as compared to other cross-sectional study done by *Medhi et al.*. (2021) which reported that the prevalence rate of diabetes mellitus among urban older adults is 23%, which might reflect rural-urban economic inequality in the prevalence of diabetes in India [30]. Barik et al. (2016) conducted a large cross-sectional study in rural West Bengal and suggested the prevalence of diabetes type 1 and 2 among adult age group below 18 years was 2.95% and 3.34% respectively [11]. And another study held in geographical variation among the diabetes prevalence elderly in India and reported southern India has a greater prevalence than north, east, and central India [31, 32]. Although our study covered a lower prevalence rate among older adults but growing number of literature suggesting risks of diabetes not only enclosed in urban areas at the same time it focused in rural areas as well [33, 34]. Considering the above fact in India more than 70% of the population belonged to rural areas, thus the growing rate of diabetes prevalence in rural areas was lower as compared to urban because the rural areas people often face issues like rural poverty, lack of health care facility, out of pocket health care expenditure in the household [10, 11, 35]. The age-specific diabetes prevalence was more common among the age groups 65–74 years. The prevalence rate of diabetes among the older adult age group was higher as compared to the younger age and it’s associated with multiple cardiovascular risk factors in the older adult age group, and delay medical treatment, low awareness, unavailability of transport facilities and lack of care are an important contributing factor for provocation of cardiovascular diseases as well as diabetes prevalence [36]. The earlier study done by Vijaykumar et al. (2009) observed a similar result [37]. Another study done by Ramachandran et al. on a high prevalence of diabetes along with NIDDM and IGT diseases among the elderly south Indian people in 2001 found that the people in the age group 60 years and above higher prevalence of diabetes and IGT as compared to the adult age group below 18 years. This study indicated that men were more likely to have diabetes than women. Weather, the previous study concluded that there have no gender differences in the prevalence of diabetes mellitus among the elderly population [10].

The present study showed that higher-educated people from wealthy economic backgrounds families had the highest prevalence of diabetes as compared to lower-educated people with the poorest economic background family. The earlier study found that educated people in north India have significantly higher treatment costs as compared to non-educated people [38]. Furthermore, data from Bangalore in 1997 and 1998 report that uneducated, unemployed people residing in semi-urban and rural areas were more likely to be treated later because of the unavailability of specialist doctors and therefore developed complications of diseases, the economic burden of the household and reduce the healthy life expectancy [39]. Similarly, currently, married people were a higher prevalence as compared to widowed which might be explained by the younger age of married participants.

Furthermore, concerning to role of lifestyle activities, the study found that adult people with not working currently were a higher prevalence of diabetes than those who are working currently. Similarly, physical activity plays a dominant role in a protective effect against diabetes, cardiovascular diseases, and metabolic syndrome which has been proven in an earlier study by Kesaniemi [40]. The study of anthropometric measurement with other controlling factors indicates a higher level of obesity inversely relationship with physical activity and has a direct impact on the risk of diabetes [9, 34]. According to Barik et al., (2016) reducing the risk of diabetes prevalence among elderly people will essentially need efforts to a healthy diet and daily physical activity [11].

The result from multivariate analysis suggested that urban older adult people have a higher risk of diabetes prevalence. The higher diabetes prevalence is associated with healthy lifestyle behavior in urban people, lack of physical activity, and increase in fat intake due to consumption of alcohol and dietary pattern [41]. The unprecedented growth of urbanization and changing urban lifestyle behavior play the dominant role for increase the diabetes prevalence among older adult people in the residence [42, 43]. Furthermore, people living in urban areas easily get access to benefits too from healthcare facilities, and the availability of transportation [44], thus lack of physical activity leads to prompt higher diabetes prevalence in urban older adults than in rural older adults respectively. The longitudinal study of diabetes type 2 was conducted in seven states in India from 1998 to 2005 and the report found that the out-of-pocket healthcare expenditure was higher in the older adult population in the urban household than in the rural counterpart [45]. This was mainly in urban areas medical consultations, laboratory tests, and treatments care are more expensive than the rural areas because these have remained unavailable. In addition, in lower income groups spending was higher in the urban than the rural population, this might be explained by a higher awareness of diabetes care among the urban poor as compared to the rural poor population [10]. Another finding reveals that the richest economic background family in rural residences were at higher risk of diabetes prevalence as compared to those who belonged to the richest quintile in urban residences [46]. However, few studies have documented the urban richest group’s preponderances of diabetes. This could be explained by the low level of awareness regarding the preventability of diabetes among the older adult. Because most rural older adults have lower education, thus the inadequate level of awareness about risk factors and the preventability of diabetes was unanticipated. In other words, wealthier people in urban residences much more spend health care expenditure in diabetes care and treatment-seeking behavior and also get better outcomes, while in rural areas relatively poorer people tend to have more difficulties accessing diabetes care and therefore spend less amount of health care expenditure and phasing difficulties of health outcomes [47]. In addition, older adult people belonging to a higher economic background family in West Bengal tend to have more treatment-seeking behavior and also more afford to pay for medical and thus leading to higher reported diabetes prevalence in this context. Another interesting finding is that lifestyle behavior fully explained the diversity of diabetes risks factor among the older adult. For example, working older adults sometimes engaged in different types of work activities and may not follow a sedentary lifestyle, which might lower the chances of diabetes prevalence as compared to those who have not done any work [48]. Furthermore, those older adult people who have used smoking, as well as smokeless tobacco and alcohol consumption and the Western dietary pattern, had a higher risk of diabetes than those who had not used any tobacco and alcohol consumption. However, this difference was not statistically significant.

### Limitations of the study

The main limitation of the study is that the study is based on self-reported it suffers from recall biases. As LASI data does not provide the laboratory experiment of Diabetes measure, therefore it cannot be directly compared with clinical data. Despite all this, the study provides reliable information on research designs and tools for long-term scientific research and awareness of Diabetes among the older adult population.

## Conclusion

In conclusion, the present study provides very relevant and vital epidemiological information regarding the high burden of diabetes mellitus among the adult older adult population in West Bengal. It has shown the unequal distribution of resources increases the trend of rural-urban differences in diabetes prevalence in the state. Although the prevalence rate is lower in rural residents compared to urban residents approximately 6% of rural older adult people suffer from diabetes millets, which indicates the prevalence of diabetes, as are important emerging issues and public health challenges in rural residents in West Bengal. Thus, the study needs policies and programs for the prevention of diabetes patients in both rural and urban areas and also make awareness of diabetes patients especially older adults through different ways such as the distribution of pamphlets, manuals, advertisements in magazines, newspapers, television, radio, and others [30]. Furthermore, make up the awareness programs designed in workplaces, public meetings, religious gatherings, schools, and colleges, especially in rural areas. In addition, need to focus on mass media and communication which would decline the rural–urban inequality in the prevalence of diabetes among older adults along with working status [48]. The basis of the findings, the study needs to make people aware and prevent diabetes by making changes to dietary habits, physical activity, and beliefs and behavior. Large-scale implementation of a National Diabetes control program and periodical diabetic screening program to be implemented to detect the disease at an early stage and prevent complications in older adults especially the 45–60 + years age group [49, 50].

## Data Availability

This research work was performed based on secondary data which is freely available upon request at the IIPS, India website (Source of data: https://www.iipsindia.ac.in/lasi).

## References

[1] Federation ID. IDF diabetes atlas 8th edition. International Diabetes Federation; 2017. pp. 905–11.

[2] Hwang CK, Han PV, Zabetian A, Ali MK, Narayan KV (2012). Rural diabetes prevalence quintuples over twenty-five years in low-and middle-income countries: a systematic review and meta-analysis. Diabetes Res Clin Pract.

[3] Hu FB (2011). Globalization of diabetes: the role of diet, lifestyle, and genes. Diabetes Care.

[4] Bishwajit G. Nutrition transition in South Asia: the emergence of non-communicable chronic diseases. F1000Research. 2015;4. 10.12688/f1000research.5732.2 10.12688/f1000research.5732.1 PMC4706051 26834976

[5] Joshi SR, Parikh RM (2007). India; the diabetes capital of the world: now heading towards hypertension. Journal-Association of Physicians of India.

[6] Saeedi, P., Petersohn, I., Salpea, P., Malanda, B., Karuranga, S., Unwin, N., … IDF Diabetes Atlas Committee. (2019). Global and regional diabetes prevalence estimates for 2019 and projections for 2030 and 2045: Results from the International Diabetes Federation Diabetes Atlas. *Diabetes research and clinical practice*, *157*, 107843. 10.1016/j.diabres.2019.107843 10.1016/j.diabres.2019.107843 31518657

[7] Shetty P (2012). Public health: India’s diabetes time bomb. Nature.

[8] Yoon, K. H., Lee, J. H., Kim, J. W., Cho, J. H., Choi, Y. H., Ko, S. H., … Son, H.Y. (2006). Epidemic obesity and type 2 diabetes in Asia. *The Lancet*, *368*(9548), 1681–1688. 10.1016/S0140-6736(06)69703-1 10.1016/S0140-6736(06)69703-1 17098087

[9] Little M, Humphries S, Patel K, Dodd W, Dewey C (2016). Factors associated with glucose tolerance, pre-diabetes, and type 2 diabetes in a rural community of south India: a cross-sectional study. Diabetol Metab Syndr.

[10] Ramachandran, A., Snehalatha, C., Kapur, A., Vijay, V., Mohan, V., Das, A. K., … Nair,J. D. (2001). High prevalence of diabetes and impaired glucose tolerance in India:National Urban Diabetes Survey. *Diabetologia*, *44*(9), 1094–1101. 10.1007/s001250100627 10.1007/s001250100627 11596662

[11] Barik A, Mazumdar S, Chowdhury A, Rai RK (2016). Physiological and behavioral risk factors of type 2 diabetes mellitus in rural India. BMJ Open Diabetes Research and Care.

[12] Ravikumar, P., Bhansali, A., Ravikiran, M., Bhansali, S., Walia, R., Shanmugasundar,G., … Dutta, P. (2011). Prevalence and risk factors of diabetes in a community-based study in North India: the Chandigarh Urban Diabetes Study (CUDS). *Diabetes & metabolism*, *37*(3), 216–221. 10.1016/j.diabet.2010.10.004 10.1016/j.diabet.2010.10.004 21195002

[13] Mohan V, Venkatraman JV, Pradeepa R (2010). Epidemiology of cardiovascular disease in type 2 diabetes: the indian scenario. J Diabetes Sci Technol.

[14] Corsi DJ, Subramanian SV (2012). Association between socioeconomic status and self-reported diabetes in India: a cross-sectional multilevel analysis. BMJ open.

[15] Meshram II, Rao MVV, Rao VS, Laxmaiah A, Polasa K (2016). Regional variation in the prevalence of overweight/obesity, hypertension and diabetes and their correlates among the adult rural population in India. Br J Nutr.

[16] Kutty VR, Dilip TR, Archana AR, Gopinathan S, Ramanathan M (2018). Shifting pattern of diabetes among the older adult in India: evidence from the national sample survey organization’s data, 2004–2014. Int J Noncommunicable Dis.

[17] Unnikrishnan AG, Sahay RK, Phadke U, Sharma SK, Shah P, Shukla R, Viswanathan V, Wangnoo SK, Singhal S, John M, Kumar A, Dharmalingam M, Jain S, Shaikh S, Verberk WJ (2022). Cardiovascular risk in newly diagnosed type 2 diabetes patients in India. PLoS ONE.

[18] Selvaraj S, Abrol D, Gopakumar KM. (2014). Access to medicines in India. *New Delhi, India: Academic Foundation*. 10.1177/0972063418799157

[19] American Diabetes Association. (2015). Classification and diagnosis of diabetes. Diabetes Care. 2015;38:s8–s16. 10.2337/dc15-S005 25537714

[20] World Health Organization. (2012). Global Health Expenditure Database. General government expenditure on health (GGHE) as % of THE. [16.10.2014]; Available from: http://apps.who.int/nha/database/Select/Indicators/en

[21] Mohan, V., Mathur, P., Deepa, R., Deepa, M., Shukla, D. K., Menon, G. R., … Shah,B. (2008). Urban rural differences in prevalence of self-reported diabetes in India—The WHO–ICMR Indian NCD risk factor surveillance. *Diabetes research and clinical practice*, *80*(1), 159–168. 10.1016/j.diabres.2007.11.018 10.1016/j.diabres.2007.11.018 18237817

[22] Jain A, Paranjape S (2013). Prevalence of type 2 diabetes mellitus in older adult in a primary care facility: an ideal facility. Indian J Endocrinol Metabol.

[23] Kirkman MS, Briscoe VJ, Clark N, Florez H, Haas LB, Halter JB (2012). Consensus report. Diabetes in Older adults Diabetes Care.

[24] Longitudinal Ageing Study in India (LASI) Wave 1, Harvard TH. International Institute for Population Sciences (IIPS), Mumbai, India, NPHCE, MoHFW. Chan School of Public Health (HSPH), and The university of Southern California (USC); 2020.

[25] American Diabetes Association (2017). American diabetes association standards of medical care in diabetes-2017. Diabetes Care.

[26] Zou D, Ye Y, Zou N, Yu J (2017). Analysis of risk factors and their interactions in type 2 diabetes mellitus: a cross-sectional survey in Guilin, China. J Diabetes Invest.

[27] Latifi SM, Karandish M, Shahbazian H, Hardani Pasand L. (2016). Incidence of prediabetes and type 2 diabetes among people aged over 20 years in ahvaz: a 5-year perspective study (2009–2014). *Journal of diabetes research*, *2016*. 10.1155/2016/4908647 10.1155/2016/4908647 PMC5149696 28004008

[28] Sinclair A, Dunning T, Rodriguez-Mañas L (2015). Diabetes in older people: new insights and remaining challenges. The lancet Diabetes & endocrinology.

[29] Luhar, S., Timæus, I. M., Jones, R., Cunningham, S., Patel, S. A., Kinra, S., … Houben,R. (2020). Forecasting the prevalence of overweight and obesity in India to 2040.*PloS one*, *15*(2), e0229438. 10.1371/journal.pone.0229438 10.1371/journal.pone.0229438 PMC7039458 32092114

[30] Medhi GK, Dutta G, Borah P, Lyngdoh M, Sarma A. Prevalence of diabetes and its relationship with body mass index among older adult people in a rural area of northeastern state of India. Cureus. 2021;13(1). 10.7759/cureus.12747 10.7759/cureus.12747 PMC7886600 33614345

[31] Gupta R, Misra A (2007). Type 2 diabetes in India: regional disparities. Br J Diabetes Vascular Disease.

[32] Gupta R, Guptha S, Sharma KK, Gupta A, Deedwania P (2012). Regional variations in cardiovascular risk factors in India: India heart watch. World J Cardiol.

[33] Deedwania PC, Gupta R, Sharma KK, Achari V, Gupta B, Maheshwari A, Gupta A (2014). High prevalence of metabolic syndrome among urban subjects in India: a multisite study. Diabetes & Metabolic Syndrome: Clinical Research & Reviews.

[34] Little M, Humphries S, Patel K, Dewey C (2016). Factors associated with BMI, underweight, overweight, and obesity among adults in a population of rural south India: a cross-sectional study. BMC Obes.

[35] Goswami AK, Gupta SK, Kalaivani M, Nongkynrih B, Pandav CS (2016). Burden of hypertension and diabetes among urban population aged ≥ 60 years in South Delhi: a community based study. J Clin Diagn research: JCDR.

[36] Gupta, A., Gupta, R., Sharma, K. K., Lodha, S., Achari, V., Asirvatham, A. J., … Deedwania,P. C. (2014). Prevalence of diabetes and cardiovascular risk factors in middle-class urban participants in India. *BMJ Open Diabetes Research and Care*, *2*(1), e000048. 10.1136/bmjdrc-2014-000048 10.1136/bmjdrc-2014-000048 PMC4256307 25489485

[37] Vijayakumar G, Arun R, Kutty VR (2009). High prevalence of type 2 diabetes mellitus and other metabolic disorders in rural Central Kerala. J Assoc Physicians India.

[38] Tharkar S, Devarajan A, Kumpatla S, Viswanathan V (2010). The socioeconomics of diabetes from a developing country: a population based cost of illness study. Diabetes Res Clin Pract.

[39] Rayappa PH, Raju KNM, Kapur A, Bjork S, Sylvest C, Kumar KD (1999). Economic cost of diabetes care: the Bangalore urban district diabetes study. Int J Diab Dev Countries.

[40] Kesaniemi YK. (2001). Dose-response issues concerning physical activity and health: an evidence-based symposium. *Med Sci Sports Exerc*, *33*, S351-S358. 10.1097/00005768-200106001-00003 11427759

[41] Muksor A, Dixit P, Varun MR. (2018). Rural-Urban Differentials in NCD Multimorbidity in Adult Population in India: Prevalence and Cost of Care. *J Trop Med Health JTMH-121. DOI*, *10*. 10.29011/JTMH-121.000121

[42] Gupta A, Belwal R, Ramakrishnan L, Khenduja P, Kapil U (2020). Association of tobacco and alcohol consumption with cardiovascular risk factors among older adult population in India. J Family Med Prim Care.

[43] Yadav K, Krishnan A (2008). Changing patterns of diet, physical activity and obesity among urban, rural and slum populations in north India. Obes Rev.

[44] Patel R, Chauhan S, Chaurasiya D, Kumar S, Paswan B (2019). Role and impact of social capital on health of older adult in India. Indian J Social Res.

[45] Yesudian CA, Grepstad M, Visintin E, Ferrario A (2014). The economic burden of diabetes in India: a review of the literature. Globalization and health.

[46] Medhi GK, Sarma J (2015). Self-rated health (SRH) among older adult diabetics in an urban setting of Assam, India. Int J Health Sci Res.

[47] Singh J. (2013). Economic burden of diabetes. *Muruganathan A, Geetha T, editors. Vol. 23. Medicine Update. Association of Physicians of India, India*, 205.

[48] Chauhan S, Srivastava S, Kumar P, Patel R (2022). Decomposing urban-rural differences in multimorbidity among older adults in India: a study based on LASI data. BMC Public Health.

[49] Vallepalli C, Kalevaru CS, Ratna B, Kumar UV, Mohan CR, Deotale PG (2020). A study of prevalence of type 2 diabetes Mellitus among adults in an Urban Population of Eluru: POT2DIE study. Indian J Public Health.

[50] Atlas D. (2015). International diabetes federation. *IDF Diabetes Atlas, 7th edn. Brussels, Belgium: International Diabetes Federation*.

